# Effects and Mechanisms of Extracellular Vesicles in Different Models of Acute Kidney Injury

**DOI:** 10.1155/sci/1075016

**Published:** 2025-03-24

**Authors:** Weidong Wang, Jingyu Wang, Dan Liao

**Affiliations:** ^1^Department of Nephrology, Mianyang Central Hospital, Mianyang 621000, China; ^2^Renal Division, Peking University First Hospital, Beijing 100080, China

**Keywords:** acute kidney injury, apoptosis, extracellular vesicles, inflammation

## Abstract

Acute kidney injury (AKI) is a rapid decline in renal function caused by ischemia/reperfusion (I/R), renal toxic injury, and sepsis. While the precise molecular mechanisms underlying AKI are still under investigation, current therapeutic approaches remain insufficient. In recent years, there has been growing evidence that mesenchymal stem cells (MSCs) have great potential in accelerating renal repair after AKI in various preclinical models, while there has been extensive research on extracellular vesicles (EVs) as therapeutic mediators in AKI models, and they are considered to be superior to MSCs as new regenerative therapies. EVs are nanoparticles secreted by various types of cells under physiological and pathological conditions. EVs derived from various sources possess biomarker potential and play crucial roles in mediating cellular communication between kidney cells and other tissue cells by transmitting signal molecules. These vesicles play a direct and indirect role in regulating the pathophysiological mechanisms of AKI and contribute to the occurrence, development, treatment, and repair of AKI. In this review, we briefly outline the essential characteristics of EVs, focus on the multiple molecular mechanisms currently involved in the protection of EVs against different types of AKI, and further discuss the potential targets of EVs from different sources in the treatment of AKI. Finally, we summarized the deficiencies in the production and treatment of EVs and the current strategies for improvement.

## 1. Introduction

Acute kidney injury (AKI) is a clinical syndrome characterized by a sharp decline in kidney function. The incidence of AKI has been gradually increasing in recent years. A previous study demonstrated that ~22% of adults and 34% of children worldwide experience AKI during hospitalization [1]. AKI is associated with a substantial mortality rate, ranging from 25% to 80%. Even patients with AKI who are not in intensive care exhibit mortality rates ranging from 10% to 20% [2]. Due to the intricate structure of the kidneys and multiple factors of kidney injury, AKI exhibits a high tendency for relapse, along with poor therapeutic outcomes, ultimately resulting in the progression of chronic kidney disease (CKD) or end-stage renal disease [3, 4]. Although many clinical trials have employed pharmacological or nonpharmacological interventions, there is a lack of reliable methods to prevent or reverse AKI. Multiple studies have substantiated the beneficial effect of mesenchymal stem cells (MSCs) in alleviating kidney cell damage [5, 6]. Extracellular vesicles (EVs) have tremendous potential as an alternative to stem cell therapy. EVs carry metabolites that are closely associated with cellular structure and function. They are internalized by neighboring or distant cells and transmit signals related to protection or injury, thus participating in the body's antigen presentation, immune response, cell differentiation, cell migration, and other processes [7, 8]. However, the role and mechanisms of EVs in renal disease have not been clarified. Therefore, we summarize the protective effects of EVs from different sources on the kidney in different experimental models of AKI. We also examine the signaling pathways through which these effects are mediated, and we explore potential therapeutic targets for AKI based on these findings. Currently, the production and mass production of EVs is still encountering challenges. The individual EVs' impact is still limited, and there is a lack of adequate utilization in diagnosing and treating AKI based on EVs. We summarize the different improvement methods for EVs.

## 2. EVs

EVs are membranous vesicles that are released into the extracellular matrix. EVs are abundant in various body fluids, such as blood, urine, saliva, and cerebrospinal fluid [9]. EVs can be divided into three subtypes according to their diameter and formation mode: EVs, microvesicles (MVs), and apoptotic bodies [10]. EVs (30–150 nm in diameter) are primarily generated through the invagination of cell membranes to form endocytic vesicles that encapsulate extracellular fluid. Subsequently, multiple endocytic vesicles merge, giving rise to multivesicular bodies (MVBs). The MVB generates intraluminal vesicles by invading the membrane. Subsequently, the MVB undergoes fusion with the cell membrane, thereby releasing the EVs into the extracellular matrix. EVs membrane has protein markers such as the expression of tetraester proteins (CD9, CD63, and CD81) and heat shock proteins (HSPs) (HSP60, HSP70, and HSP90). MVs (200–1000 nm in diameter) are produced by the fission of the plasma membrane into the outgoing bud and the subsequent release of vesicles into the extracellular space. Apoptotic bodies (800–5000 nm in diameter) are generated during the apoptotic process [11]. The plasma membrane undergoes intricate folding, expansion, division, and cytoplasmic wrapping, forming vesicles that encompass DNA materials and organelles. In EV production, the composition of liquid and solid is different due to the different sizes of vesicles. Moreover, the protein, nucleic acid, and metabolic substances found in EVs may not be entirely identical to those in mother cells due to various microenvironments and sources of mother cells [12]. These findings were confirmed by Wang et al. [13], who discovered that HK-2 cells possess 421 lipids and 218 EVs, with 205 lipids being shared between cells and EVs. Saito et al. [14] detected 855, 763, and 396 metabolites in renal tissue, urine, and EVs, respectively. A total of 325 metabolites overlapped, including 280, 207, and 12 metabolites specific to renal tissue, urine, and urine EVs, respectively [14]. Due to differences in metabolites, EVs interact with target cells through multiple mechanisms, facilitating intercellular information transfer and governing autophagy, apoptosis, inflammatory responses, and proliferation of kidney cells [15–17]. In addition, there are distinct differences in the composition of EVs secreted by cells under pathological and physiological conditions. Certain miRNAs or groups of miRNAs, proteins, and metabolites present in EVs can potentially be utilized for early diagnosis, disease staging, and prognostic assessment of various kidney, liver, heart, and other organ diseases [18–22]. In addition, EVs can play a role in the treatment of kidney disease as therapeutic agents and drug carriers. As a carrier, EVs have many advantages compared with other nanomaterials, including improved stability, biocompatibility, low immunogenicity, enhanced permeability across biological barriers, and reduced toxicity. This review summarizes EVs' therapeutic effects and potential role from different sources in AKI induced by different factors.

## 3. AKI Induced by Ischemia/Reperfusion (I/R)

I/R is characterized by structural changes and functional injuries in organs that result from either ischemia or the restoration of blood perfusion for various reasons. When ischemia occurs, the accumulation of reactive oxygen species (ROS) in the body hinders the transfer mitochondrial transcription factor A (TFAM) mRNA transcription and facilitates the release of TFAM and mitochondrial DNA (mtDNA) into the cytosol. TFAM acts as a pivotal regulator for initiating mtDNA transcription, and its absence further diminishes mtDNA synthesis and biogenesis, thereby inducing depletion of mtDNA and respiratory defects during I/R injury-associated AKI (IRI-AKI) [23]. Zhao et al. [24] have shown that MSCs-EVs transfer TFAM contained in MSCs-EV to maintain the stability of TFAM protein and TFAM–mtDNA complex. Overexpression of TFAM can partially ameliorate kidney damage [24]. However, previous studies have also demonstrated that TFAM overexpression leads to an increase in mtDNA without a concomitant rise in mitochondrial respiratory chain content or total mitochondrial abundance [25]. Consequently, the beneficial effects of TFAM EVs on kidney injury do not stem from an augmentation in mitochondrial numbers. A recent proposal suggests that during oxidative stress or inflammatory responses, TFAM interacts with autophagy protein LC3B, serving as an autophagy receptor to clear abnormally located cytosolic mtDNA, thereby inhibiting the cGAS-STING pathway and alleviating the inflammatory response triggered by mtDNA [26]. The respiratory factor 2, acting as the proximal promoter of TFAM, has been demonstrated to mitigate renal injury induced by oxidative stress through the regulation of EVs [27]. Cao et al. [28] further substantiated that the potential mechanism involves EVs-derived miRNA-200a-3 reducing mitochondrial damage, restoring mitochondrial membrane potential, and increasing the number of mtDNA copies by targeting the Keap1-Nrf2 signaling pathway. Currently, it has been confirmed that EVs-derived miRNA-20a-5p exerts a protective effect on the kidney. However, its regulatory mechanism remains unclear and may be associated with the Nrf2/TFAM pathway, which requires further clarification through extensive experimentation [29]. Additionally, EVs loaded miR-30 has the ability to regulate mitochondrial fission through dynamin-related-protein 1 (DRP1) in order to alleviate mitochondrial fragmentation [30]. Lopes et al. [31] have shown that EVs derived from adipose tissue mesenchymal stromal cells (ATMSCs) can partially reverse the injury of proximal renal tubules caused by hypoxia by inhibiting the formation of mitochondrial O2-1 and upregulating the antioxidant HO–1/nuclear factor erythroid 2-related factor 2 system.

Numerous studies have demonstrated that acute I/R in any tissue results in a rapid and robust inflammatory response characterized by the recruitment of inflammatory cells, cytokine production, generation of free radicals, and induction of oxidative stress [32, 33]. During early reperfusion, M1 macrophages are first activated to induce renal tissue damage by producing excess ROS, nitrogen intermediates, and proinflammatory cytokines such as interleukin-1*β* (IL-1*β*) and tumor necrosis factor-*α* (TNF-*α*) [34, 35]. EVs can ameliorate inflammatory damage by inhibiting macrophage activation [36]. The mechanism is that MSC-EV upregulates the C─C motif chemokine receptor 2 (CCR2), effectively reduces the level of free CCL2 and inhibits its ability to recruit or activate macrophages [37]. EVs-derived miR-146a-5p has been shown by Li et al. [38] to inhibit interleukin-1 receptor-associated kinase 1 (IRAK1) expression and nuclear translocation of the NF-*κ*B p65 subunit, thereby exerting an inhibitory effect on inflammation through targeting IRAK1′s 3′ UTR. In addition to the above mechanisms, tubular epithelial cells (TECs)-EVs CD26p inhibits the inflammatory expression by suppressing the downstream activity of chemokine and stroma-derived factor-1 (SDF1) mediated by the G protein-coupled receptor C–X–C chemokine receptor type 4 (CXCR4). Besides, TECs-EVs CD26p inhibited the expression of excess collagen I in I/R, thereby preventing the progression of AKI to CKD [39]. A substantial body of research has demonstrated significant disparities in miRNA composition between EVs derived from normal cells and those derived from AKI. These distinct miRNAs are known to play a crucial role in the activation of M1. MiRNA-23a-rich EVs target A20 in vitro, thereby promoting the activation of macrophages [40]. MiR-19b-3p, miR-374b-5p, and miR-155 within the EVs, which are directly implicated in the modulation of cytokine signaling 1 (Socs1), activation of MI macrophages, and subsequent stimulation of the NF-*κ*B pathway [41–43]. It is unclear whether these miRNAs bind to Socs1 at different sites or at the same site and whether they are regulated by other factors. However, it is evident that EV-derived miRNAs have a dual effect on renal IRI, both promoting inflammation and inhibiting the inflammatory response. Further research is needed to determine which miRNAs are proinflammatory and which are anti-inflammatory.

I/R leads to programed cell necrosis, including apoptosis, autophagy, and necrosis. Previous studies have shown that EVs can promote proliferation, inhibit apoptosis, and regulate autophagy. Abundant miR-125b-5p in MSC-EVs inhibits p53 protein expression in TEC while increasing cyclin-dependent kinases 1 (CDK1) and Cyclin B1 levels, thus alleviating G2/M blockages and promoting proliferation. Furthermore, it was found that regulating B-cell lymphoma-2 (Bcl-2) and bcl-2-related X (Bax) expression levels effectively inhibited the apoptosis of TEC [44]. MiR-216-5p in USC-EVs results in decreased expression of phosphatase and tensin homolog (PTEN) and promotes Akt phosphorylation [45]. Similarly, miR-486-5p in human cord blood endothelial colony-forming cell (ECFC)-derived EVs also modulates this pathway [46]. Additionally, Vinas et al. [47] further validated that the interaction between CXCR4 and endothelial SDF-1*α* facilitates the efficient transportation of miR-486-5p within EVs. EVs derived from human bone marrow MSCs (hBMSCs) containing MiR-199a-3p have been shown to mitigate oxidative stress-induced damage by regulating the Sema3A/Akt/extracellular signal-regulated kinase (ERK) pathway [48]. Additionally, the microRNA (miR)-199a family member, Mir-199A-5p, has been found to interact with BIP to inhibit I/R-induced endoplasmic reticulum (ER) stress and alleviate AKI [49]. Moreover, MSC-EVs treatment during I/R injury inhibited necrotic apoptosis by activating the ERK 1/2 phosphorylation signaling pathway [50]. Yuan et al. [51] iPSC-derived MSCs (iMSCs) derived EVs could be directly potentiated. The transfer of SP1 into HK-2 cells increases SP1 expression in receptor cells and activates the sphingosine kinase signaling pathway (SP1/SK1/S1P) in renal tubular epithelium. This activation attenuates the necrotic apoptosis of renal TECs and inhibits the intrinsic immune response triggered by damage-associated molecular patterns (DAMPs) [51]. Zhang et al. [52] found that EVs with overexpression of Oct-4 in the treatment of AKI could significantly reduce serum creatinine and urea. However, the specific mechanism underlying this effect requires further investigation [52]. Chen et al. [53] demonstrated that following cardiac surgery, the level of miR-590-3p in EVs increased in both the young and old cohorts, with a statistically significant increase observed in the young group. Elevated miR-590-3p has been shown to mitigate I/R injury through paracrine signaling. However, the specific target of miR-590-3p in this process remains unclear. Through bioinformatics analysis, Chen et al. [53] identified TRAF6 as a potential target gene of miR-590-3p, which holds great significance for the management of postsurgical AKI patients. However, the identification of miRNAs derived from EVs remains limited, and further extensive experimentation is required to fully explore the effects and targets of miRNAs in this context. The above mechanism is summarized in Table 1.

## 4. Cisplatin-Induced AKI

Cisplatin is commonly used as a chemotherapy drug for treating various solid tumors in clinical practice. Cisplatin is associated with nephrotoxicity, and ~30%–40% of patients undergoing cisplatin treatment may develop AKI [54]. There is a lack of effective drugs for cisplatin-induced AKI, with the exception of kidney replacement therapy [55].

Currently, EVs have been found to be a novel treatment for cisplatin-induced AKI. Stem cells from the apical papilla (SCAPs)-derived EVs have been found to affect cisplatin-treated NRK-52E cells, increasing the survival rate of damaged cells from 65% to 89%. The ROS decreased from 176% to 123%. Glutathione levels exhibited a significant increase of 78%, whereas the levels of malondialdehyde and TNF-*α* showed a decrease of 35% and 9%, respectively. In addition, they can also reduce the expression of NF-*κ*B, IL-1*β*, and p53 inflammatory factors in AKI. At the same time, the gene expression of antiapoptotic factor Bcl-2 was increased, and the gene expression of pro-apoptotic factor Bax and caspase-8 (CASP8), CASP9, and CASP3 were decreased [56].

Wang et al. studied that Let-7b-5p in MSC-EVs reduced apoptosis in renal tubule epithelium cells by inhibiting P53 expression, thereby reducing DNA damage and apoptosis pathway activity. In summary, a decrease in Let-7b-5p may lead to an increase in kidney AKI susceptibility, and in addition, an upward adjustment of the aging-related DNA damage/P53 pathway may be an important predisposing factor for cisplatin-induced AKI [57]. hucMSC-EVs can enhance cell proliferation by activating ERK 1/2 pathway [58], miR8114 in MSC-EVs can reverse the G1 phase arrest of HK2 cells and alleviate cisplatin-induced growth inhibition by regulating the expression of forkhead box O4 (FOXO4), p27 Kip1 and CDK2 proteins [59].

Wang et al. [60] discovered that EVs derived from human umbilical cord MSCs (hucMSCs-EVs) regulated the expression of the autophagy marker protein LC3B and autophagy-related genes ATG5 and ATG7, leading to the activation of autophagy. They also found evidence suggesting that this regulation may be achieved through the inhibition of the mTOR signaling pathway [60]. Wang et al. [61] 14-3-3*ζ* transported by hucMSC- EVs may upregulate the autophagy level of HK-2 cells. No previous studies have demonstrated a regulatory effect of 14-3-3*ζ* on the mTOR pathway. Therefore, the correlation between EVs derived 14-3-3*ζ* and the mTOR pathway remains unclear. In addition, haec-EVs can effectively inhibit the TNF-*α*/MAPK signaling pathway, showing its potential as an anti-inflammatory agent. In addition, in cisplatin treated kidneys, EVs have anti-inflammatory effects while neither stimulating tumor growth nor interfering with the therapeutic effect of cisplatin. This provides a new idea for the prophylactic use of EVs in cisplatin therapy [62]. Ma et al. [63] found that cisplatin-induced AKI in mice can be alleviated by downregulating mir30a-5p in urine EVs of premature infants through MAPK8 [63]. These studies suggest that EVs can be regulated in cisplatin-induced AKI through multiple pathways. However, further exploration is needed to understand the detailed mechanisms behind these effects. The role of EVs in the association of cisplatin-induced AKI is summarized in Table 2.

## 5. Sepsis-Associated AKI (S-AKI)

It is well known that sepsis is a significant factor and essential inducer of AKI in critical patients [64]. Recent studies have revealed that the mechanisms of S-AKI extend beyond decreased renal perfusion and secondary renal TEC death. Furthermore, inflammation, dysfunction of microcirculation, and metabolic reprograming also play crucial roles [65–67]. Zhang et al. [68] investigated the effects of EVs derived from adipose tissue MSCs and bone marrow MSCs on improving renal function injury induced by LPS, focusing on reducing oxidative stress and inflammatory response. Compared with bone marrow-derived MSC EVs, adipose-derived MSC (ADMSC) EVs significantly improved renal function and structure [68]. During LPS-induced sepsis, activation of the inflammatory response and increased expression of inflammatory factors lead to the promotion of monocyte migration and adhesion to vascular endothelium by IL-8 and MCP-1, which results in the extravasation of monocytes into the surrounding tissue, ultimately leading to kidney tissue damage. Human amniotic epithelial cell (hAEC)-derived EVs have been shown to inhibit the activation of the NF-*κ*B pathway, reduce the expression of inflammatory factors IL-1*β*, TNF-*α*, IL6, and IL8, as well as maintain the adhesion of endothelial cells (ECs). This helps to preserve the integrity of kidney endothelium and reduce inflammatory exudation, ultimately leading to a reduction in kidney damage caused by CLP [69]. Similar findings were also observed in a study involving EVs derived from adipose tissue-derived MSCs (AMSCs). Furthermore, it has been discovered that hypoxic-preconditioned muscle ducts transport miR-21 from preischemic limbs to distal organs. Subsequently, miR-21 targets downstream programed cell death 4 (PDCD4)/NF-*κ*B and PTEN/AKT pathways, demonstrating anti-inflammatory and antiapoptotic effects [70]. Bian et al. [71] also discovered that EVs inhibit the activation of the NF-kB/NLRP3 pathway through the miR-21-5p/TLR4 axis. This continuous inhibition effectively reduces inflammation and prevents TEC pyroptosis in AKI mice while also promoting TEC repair [71]. Endothelial progenitor cells (EPCs)-derived EVs miR-21-5p has been found to silence RUNX1 in order to reduce kidney injury and regulate intrarenal injury markers syndan-1 and heparinase-1. This results in decreased expression levels of ET-1, iNOS, TNF-*α*, and IL6, while increasing MDA levels, inhibiting SOD activity, leads to a reduction in septic-induced oxidative stress [72]. These effects may be attributed to the activation of SIRT1 by EVs [73]. The Sirtl gene acts as a substrate for NF-*κ*B and contributes to inflammation reduction through its deacetylation process [74]. Previous studies have indicated that miR-21 plays a regulatory role on Sirtl [75]; therefore, miRNA 21 within EVs may exert an anti-inflammatory effect through Sirtl regulation. Sirtl is also a key factor in renal tubule apoptosis closely related to pro-apoptotic genes Bax and Bim and antiapoptotic genes Bcl2 and Bcl-Xl [76, 77]. However, no relationship between Sirtl and these apoptosis-related molecules has been observed. Studies have found that Sirtl regulates apoptosis by deacetylating these transcription factors, such as p53, FoxOl, and FoxO3a [78]. However, it remains unknown whether EVs-derived miRNA 21 achieves its effects through the regulation of Sirtl. It has been confirmed that Sirt1 plays a crucial role in the regulation of sepsis-associated inflammatory injury and apoptosis in EVs. Therefore, Sirt1 is considered to be a highly promising target for further research and potential therapeutic interventions. Excepting miRNA 21, Zhang et al. [79] observed that hucMSC-EVs decreased the expression of IRAK1 in renal tubule cells by upregulating the level of miR-146b, inhibited the activation of NF-*κ*B induced by CLP. EVs miR-93-5p derived from EVS and its derivatives directly affect lysine (K)-specific demethylase 6B (KDM6B), leading to increased expression of histone H3 Lys27 trimethylation (H3K27me3) and a significant reduction in TNF-*α* content, which can inhibit LPS-induced inflammation and apoptosis of HK2 cells and reduce vascular leakage and organ damage [80]. Previous studies have pointed out that macrophages play important roles in the progress of AKI. The odds ratio between M1 and M2 determines the self-repair process of AKI [81, 82]. The expression levels of miRNA in M1 and M2 EVs were different. Juan et al. [83] showed that there are differences in miRNA expression levels in M1 and M2 EVs, both of which exhibit the characteristics of their respective mother cells. M1 EVs induced the release of IL-1*β* and IL-18 and promoted the expression of pyroptosis-related proteins. M2-derived EVs may alleviate the above-mentioned injuries. There are significant differences in the expression of miR-93-5p in M1 and M2 EVs, suggesting that miR-93-5p may play a role in regulating liPS-induced pyroptosis through EVs. Additionally, bioinformatics prediction and luciferase reporter gene analysis revealed thioredoxin interacting protein (TXNIP) as a direct target of miR-93-5p. Therefore, it is hypothesized that M2-derived miR-93-5p mitigates renal tubular cell pyroptosis by modulating TXNIP [83]. The mechanism of EVs in S-AKI is summarized in Table 3.

## 6. Glycerol-Induced AKI

In glycerol-induced AKI, the regeneration of EVs has get more attention [84]. Bruno et al. [85] discovered that BMMSC-EVs, particularly exosomes, contain high levels of specific mRNA (CCNB1, CDK8, and CDC6) that impact the initiation and advancement of cell cycles. The increased miRNAs encourage proliferation through growth factors (HGF and IGF-1), thereby alleviating AKI [85]. Besides, EVs modulate the metabolic pathways associated with fatty acid oxidation, glycolysis, gluconeogenesis, and ketone body production in damaged TECs by delivering miRNAs to enhance the expression of genes involved in these pathways, restoring the kidney injury induced by glycerol [86]. Grange et al. [87] have discovered a renal protective factor known as urinary EVs carrying Klotho, a protein primarily synthesized in the kidneys and involved in renal homeostasis, physiology, and pathology. This factor has the ability to specifically target damaged kidneys and promote regeneration [87]. Chen and Hou [88] found that glycerol-induced rhabdomyolysis induced AKI. BMSCs coincubated with SPION produce magnetic exosomes (Exo-SPION), which lead to the direct targeting of damaged muscle by an external magnetic field, upregulating the ERK signaling pathway, thereby regulating the expression of various repair factors. At the same time, collagen synthesis can be promoted by inhibiting the WNT/b-catenin signaling pathway, which can slow down the conversion of AKI to CKD [88].

## 7. Folic Acid (FA)-Induced AKI

Low doses of FA confer nutritional benefits, whereas high doses are highly nephrotoxic. Previous studies have demonstrated that FA can induce AKI via mechanisms such as oxidative stress, ferroptosis, mitochondrial dysfunction, elevated levels of fibroblast growth factor 23 (FGF23), impaired mitochondrial autophagy, and others [89]. Yu et al. [90] discovered that exosomes exacerbate kidney injury. Mice treated with myofibroblast-derived exosomes (Myo-Exos) exhibited severe fibrotic lesions and an increased transition of macrophages into myofibroblasts (MMTs) in kidneys subjected to FA-induced injury. Inhibition of exosome production mitigated collagen deposition, extracellular matrix protein accumulation, and MMT in FA-induced nephropathy [90].

## 8. Modification of EVs

**Figure 1:**
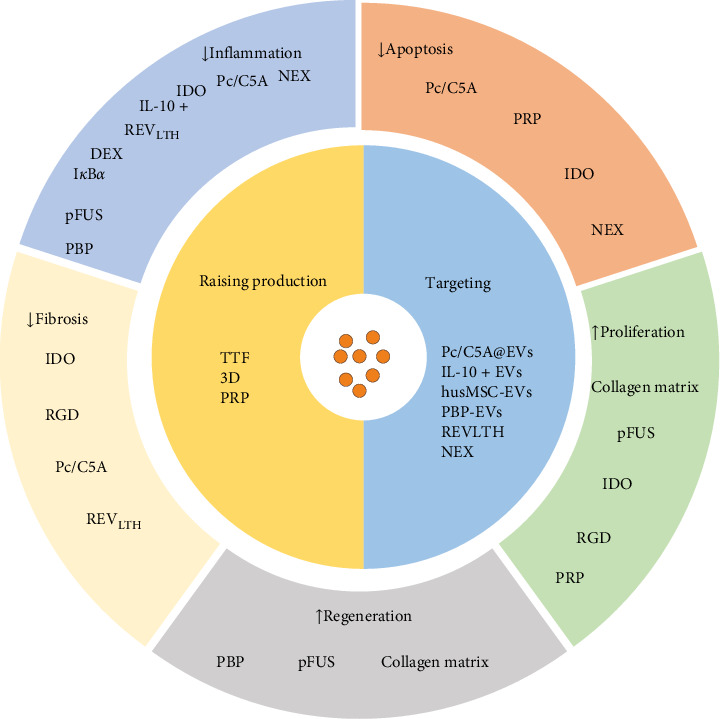
Modification of extracellular vesicles by different substances.

Stem cell-derived EVs therapy is a highly promising treatment strategy for AKI. The long regeneration times, short half-lives of in vivo EVs, and their low retention and stability after transplantation impose limitations on their potential clinical applications. Therefore, it is essential for EVs optimization (Figure 1). To address the issue of EVs production, Lee et al. [91] proposed that tangential flow filtration (TFF) technology could efficiently generate a substantial quantity of EVs from human AMSCs [91]. After generating a large number of EVs using the TTF technology, the I*κ*B*α* is loaded into EVs (EVs-I*κ*B*α*) relying on the Optoge network-engineered EVs system. The size of EVs-I*κ*B*α* generated by the above method is consistent with EVs. Moreover, EVs-I*κ*B*α* may reduce the sepsis-mediated inflammatory response by inhibiting the NF-*κ*B pathway [92]. Kang et al. [93] developed a new method to produce using a flat-plate bioreactor, which produced approximately seven times more EVs per cell than that under static conditions within a day. The EVs also maintain the MSC characterization and enhance EV efficiency [93]. Zhang et al. observed that a three-dimensional (3D) culture could generate more EVs than a traditional monolayer culture (2D) [94]. The same conclusion was also reached in human placental MSCs (hPMSCs)-derived EVs. Moreover, the 3D-cultured EVs derived from hpMSCs effectively suppress the apoptosis of renal TECs induced by I/R and decrease the generation of proinflammatory factors [95]. Lin et al. [96] found that EVs derived from ADMSCs exhibit reparative effects on DNA damage, apoptosis, fibrosis, and glomerular and tubular injury induced by I/R. However, treatment with ADMSC in combination with ADMSC-derived EVs demonstrates superior efficacy compared to either therapy alone [96]. Pretreating with small-molecule drugs is a commonly utilized strategy to enhance the efficacy of stem cell transplantation. Ji et al. [97] found that treatment of MSCs with platelet-rich plasma (PRP) resulted in a twofold increase in EVs production while not altering the morphology and size of MSC-EVs. In addition, PRP stimulates the paracrine activity of MSCs EVs by activating the AKT/Rab27 pathway, inhibiting apoptosis in renal tubule cells and promoting recovery of renal function [97]. Therapeutically, surface ligands such as peptides can be loaded onto EVs to confer specific targeting capabilities and enhance their functionality. Cheng et al. [98] developed a novel nano-sized hypoxia-sensitive coassembly (Pc/C5A@EVs). Msc-EVs directed Pc/C5A to hypoxic kidneys via adhesion molecule integrin receptors *α*4*β*1 and *α*L*β*2. Pc/C5A@ EVs can induce the transformation of M1 macrophages into M2 macrophages by inhibiting the expression of hypoxia-inducible factor-1*α* (HIF-1*α*), and NF-*κ*B signaling pathway in TECs, alleviates renal tubulointerstitial inflammation and restores renal function [98]. Xie et al. [99] showed that bone marrow MSC-derived EVs with indoleamine 2,3-dioxygenase (MSC-EVs-IDO) could change the inflammatory microenvironment of renal tubule cells by promoting the polarization of M1 macrophages to M2 macrophages, thus promoting the self-repair process of mice after IRI, and its therapeutic effect is significantly better than that of MSCs EVs alone. Tang et al. [100] found that when IL-10 is equipped on EVs, IL-10+ EVs enhance the stability and targeting of IL-10. They also promote mitochondrial phagocytosis to maintain mitochondrial homeostasis of TECs by inhibiting the mTOR signaling pathway. Moreover, IL-10+ EVs drive the polarization of tubulointerstitial macrophages, thereby improving the repair of kidney injury caused by I/R [100]. Dexamethasone is loaded onto macrophage-derived EVs (MV-DEX), delivering glucocorticoid receptors to kidney cells. This process increases the cellular levels of the receptor, enhancing cell sensitivity to glucocorticoids and leading to improved renal protection and anti-inflammatory effects in mice treated with LPS. Furthermore, it was found that the delivery of DEX through MV was more effective than direct treatment with dexamethasone. Significantly, MV-DEX alleviated the adverse effects of glucocorticoid therapy, including hyperglycemia and suppression of the hypothalamic–pituitary–adrenal (HPA) axis [101].

Liu et al. [102] demonstrated that coating EVs with a collagen matrix enhances their stability, facilitates sustained release, and prolongs them in vivo activity. Compared to using EVs alone, applying collagen matrix significantly promoted proliferation and angiogenesis in renal TECs, inhibited apoptosis in renal cells, and improved fibrosis in AKI by inhibiting ER stress [102]. Zhang et al. [103] developed a biocompatible type of arginine-glycine-aspartate (RGD) peptides. The RGD can be combined with the MSC-EV membrane surface to improve the retention and stability of EVs, promoting the bioavailability of EVs. Further studies have found that the combination of RGD hydrogel and EVs amplifies and extends the protection of EVs against ischemic AKI. The mechanism is that RGD promotes MSC-EVs containing microRNA let-7a-5p to inhibit cell apoptosis by reducing CASP3 expression and activating autophagy activation by downregulating the amino acid sensing pathway [103]. In a cisplatin-induced AKI model, Ullah et al. [104] found that EVs could inhibit HSP70/90, reducing the expression of NLRP3 and downstream proinflammatory cytokines. As a result, EVs were able to alleviate AKI. Pulsed-focused ultrasound (pFUS) can enhance the ability of EVs to treat AKI [104]. Further studies have found that the main molecular mechanisms of EVs combined with pFUS treatment involve the modulation of MAPK/ERK and PI3K/Akt signaling pathways, SIRT3 and eNOS pathways, as well as the suppression of inflammation. Although the combination of pFUS and EVs can enhance therapeutic efficacy, pFUS does not enhance the homing of EVs [105]. Wu et al. [106] engineered hybrid vesicles (NEX) by constructing small EVs derived from the human neutrophil cell membrane and human umbilical cord MSCs and then fused them with hucMSCs-EVs. NEX significantly enhances the targeting ability of hucMSC-EVs to damaged kidney tissue and inhibits phagocytosis by macrophages. Furthermore, this intervention may ameliorate cisplatin-induced AKI by anti-inflammation, promoting proliferation, and inhibiting apoptosis. Nevertheless, the specific underlying mechanism needs further investigation [106]. Zhang et al. utilized hydrophobic insertion to modify the P-selectin binding peptide (PBP) on the EV surface, creating PBP-engineered EVs (PBP-EVs) with the capacity to specifically recognize and bind P-selectin on injured ECs. Targeting the binding to damaged ECs can reduce inflammatory cell infiltration and increase reparative angiogenesis in injured kidneys after severe IRI. There is a positive correlation between Cy5.5 radiation and kidney injury in PBP-EVs. Therefore, PBP-EVs could serve as a more precise indicator of the severity of kidney injury compared to general EVs [107]. Tang et al. [108] loaded targeting peptides and therapeutic siRNAs (LTH) on red blood cell-derived EVs (REVs). Targeted delivery of siRNA against P65 and Snai1 effectively inhibited the expression of P-p65 and Snai1 in renal tubules, leading to reduced tubulointerstitial inflammation and fibrosis, significantly ameliorating I/R- and UUO-induced renal injury and effectively blocking the progression of AKI to CKD [108].

### 8.1. EVs as Biomarkers

Currently, the assessment of renal injury primarily depends on measuring serum creatinine levels. However, creatinine exhibits limited sensitivity and specificity in detecting or classifying the severity of AKI. Moreover, it needs to catch up to the actual occurrence of renal damage and the subsequent recovery [109, 110]. Recent studies have demonstrated the potential for early detection of AKI through analysis of EVs components, allowing for precise determination of the degree and type of renal damage. This approach holds promise for achieving timely diagnosis and intervention in AKI cases [111, 112]. Sonoda et al. [113] found that the release of uEV-AQP1 significantly increased 24 h after cisplatin-induced AKI and then gradually returned to the baseline levels. The release of uEV-AQP1 is significantly reduced at 168 h. In contrast, a reversed trend is observed in the release of uEV-AQP2. The results suggest that the release of uEV-AQP2 could serve as an indicator for evaluating renal impairment at different stages of cisplatin stimulation, from early to late. In addition, the combination of uEV-AQP2 and -AQP1 may be useful in estimating cisplatin-induced renal injury in a stage-dependent manner [113]. Yu et al. [114] found that urinary EVs derived NHE3 was increased in AKI caused by various factors, such as cisplatin treatment, furosemide injection induced by a low-NaCl diet, IRI, and sepsis. Furthermore, urinary EVs derived NHE3 increased on the second day in rats with cisplatin-induced AKI 1 day before the elevation of serum creatinine and blood urea nitrogen (BUN). Urinary EVs derived NHE3 also increased earlier than serum creatinine in the low NaCl diet and candesartan-associated AKI setting. In other rats, urinary EVs derived NHE3 decreased with SCR after EPO pretreatment. Therefore, it has high sensitivity as a diagnostic biomarker for AKI [114]. Miller et al. [115] indicated that AKI patients had a significant reduction in podocarin-positive EVs before cardiac surgery, and their levels correlated with the injury's severity. Hence, preoperative podocarin-positive EVs levels are predictive for AKI [115]. Awdishu identified 251 dysregulated proteins in the EVs-derived proteins obtained from patients with vancomycin-associated AKI (V-AKI) compared to healthy controls. Proteins in these EVs may serve as biomarkers for drug-induced disease processes. However, additional investigations are required to validate this hypothesis [116]. The AKI cohort study revealed a significant inverse correlation between levels of urinary EVs derived CD26 and the occurrence of adverse renal events within 90 days. Additionally, a positive correlation was observed between these levels and renal repair. Patients with high cd26 levels had an earlier reversal and significantly higher reversal rates than patients with low cd26 levels. In contrast to non-AKI patients, urinary EVs-derived CD26 was negatively correlated with non-AKI patients. Therefore, the above evidence suggests that urinary EVs derived l CD26 level is an important indicator of kidney-related prognosis and has predictive value for early reversal of AKI [117].

## 9. Conclusion

This review mainly summarizes the protective effects of EVs from different sources on different forms of AKI. A growing number of experiments have shown that EVs derived from different types of cells exert direct or indirect effects on renal cells. The main mechanisms include regulating renal cell proliferation, autophagy, apoptosis, oxidative stress, and inflammatory response. EVs have great potential for diagnosis and prognosis assessment of different types of AKI. However, EVs still have limitations in clinical applications. The targeting, stability, and functionality of EVs can be enhanced through various approaches, including surface modification, genetic engineering, and nanoparticle hybridization. While EVs from various sources show promising prospects in the treatment of AKI in both in vivo and in vitro experiments, their clinical application requires further comprehensive research.

## Figures and Tables

**Table 1:** Functional pathway of EVs in AKI induced by I/R.

EVs of source	Model (vitro/vivo)	Pathway	Mechanism	Reference
ATMCs	Rats/HK-2 cell	HO-1/nuclear factor erythroid2-related2 system	↓ Apoptosis ↓ Mitochondria injury	[31]
MSCs	Mice/HK-2 cell	miR-200a-3p/keap1-Nrf2	↑Mitochondria function	[28]
MSCs	Mice/HK-2 cell	TFAM and mtDNA abondance	↓ Oxidative stress ↓ Mitochondria damage	[24]
MSCs	Rats	miR-30/DKP1	↓ Mitochondria apoptosis	[30]
Hy	Mice	miR-20a-5p	↓ Apoptosis ↓ Mitochondria damage	[29]
MSCs	Rats	NF-*κ*B	↓ Apoptosis ↓ Inflammation	[36]
MSCs	Mice/macrophage	CCR2/CCL2	↓ Inflammation	[37]
USCs	Rats	Caspase-9 miR146a-5p/IRAK1/NF-*κ*B	↓ Apoptosis ↓ Inflammation	[38]
TECs	Mice	CD26/p53/p21	↑Proliferation ↓Inflammation ↓Fibrosis	[39]
MSCs	HK-2 cell	miR-125b-5p/p53	↓Apoptosis ↓G2/marrest	[44]
USC	HK-2 cell	miR-216-5p/PTEN/Akt	↓Apoptosis	[45]
ECFC	Mice	CXCR/SDF-1*α*/miR-486-5p/PTEN	↓Apoptosis	[46, 47]
hBMSCs	HK-2 cell	MiR-199a-3p/Sema3A/Akt ERK	↓Apoptosis	[48]
BMSCs	Mice	miR-199a-5p/BIP	↓ER stress	[49]
hiPSC	Rats/HK-2 cell	SP1/SK1/S1P	↓Necroptosis	[51]
iMSC-Exo	Mice	ERK1/2	↓Apoptosis	[50]
MSC	Mice	Oct-4 Oct-4/snail gene	↓Apoptosis ↑Proliferation ↓Fibrosis	[52]
HK-2	HK-2 cell	miR590-3P/TRAF6	↑Autophagy	[53]

**Table 2:** Functional pathway of EVs in cisplatin-induced AKI.

EVs of source	Model	Pathway	Mechanism	Reference
The apical papilla	Cisplatin	Caspase signal NF-*κ*B signal ROS/GSH/MDA/TNF*α*	↓Apoptosis ↓Inflammation ↓Oxidative stress	[56]
MSC	Cisplatin	Let-7b-5p	↓Apoptosis ↓DNA damage	[57]
HucMSC	Cisplatin	LC3B/ATG5/ATG7	↑Autophagy	[60]
HucMSC	Cisplatin	14-3-3*ζ*	↑Autophagy	[61]
HucMSC	Cisplatin	MAPK PCNA/ERK1/2	↓Oxidative stress ↓Apoptosis ↑Proliferation	[58]
hAECs	Cisplatin	TNF*α*/MAPK Caspase signal	↓Inflammation ↓Apoptosis	[62]
MSC	Cisplatin	MiR-1184/FOX4/P27/kipl/CDK2	↓Growth inhibition	[59]
Urine	Cisplatin	miR-30a-5p/MAPK8	↓Apoptosis	[63]

**Table 3:** Functional pathway of EVs in sepsis-associated AKI.

EVs of source	Model	Pathway	Mechanism	Reference
hAECs	CLP	NF-*κ*B	↓Inflammation ↓Endothelial dysfunction	[73]
Mouse serum Human serum Supernatant of myotubes	CLP	MiR-21/PDCD4/NF-*κ*B MiR-21/PTEN/AKT	↓Inflammation ↓Apoptosis	[70]
EPCs	CLP	MiR-21-5P/Runx1	↓Inflammation ↓Apoptosis ↓Oxidative stress	[72]
ADSC	LPS	MiR-21-5p/NF-*κ*B/NLRP3	↓Pyroptosis ↓Inflammation	[71]
EPCs	LPS	MiR-93-5p/KDM6B /H3K27me3/TNF-*α*	↓Inflammation ↓Apoptosis ↓Vascular leakage	[80]
M2 macrophage	CLP	MiR-93-5P/TXNLP /NLP3	↓Pyroptosis	[83]
hucMSC	CLP	MiR-146/IRAK1/ NF-*κ*B	↓Inflammation	[79]

## Data Availability

Research data are not shared.
